# Historical Redlining and Spatiotemporal Patterns in Breast Cancer Screening

**DOI:** 10.1001/jamanetworkopen.2026.30685

**Published:** 2026-09-02

**Authors:** Md Atikur Rahman, Isuru Ratnayake, Sam Pepper, Derrick Asante, Brahian Cano Urrego, Susan Beckman, Leah Lambart, Dinesh Pal Mudaranthakam

**Affiliations:** 1 Department of Biostatistics and Data Science, University of Kansas Medical Center, Kansas City; 2 The University of Kansas Cancer Center, University of Kansas Medical Center, Kansas City; 3 Masonic Cancer Alliance, University of Kansas Cancer Center, Fairway

## Abstract

**Importance:**

Geographic variation in breast cancer screening may reflect persistent structural inequities in access to preventive care. Understanding whether historical redlining remains associated with screening, independent of contemporary social vulnerability, neighborhood conditions, and geographic access, is critical for targeting interventions within cancer center catchment areas.

**Objectives:**

To examine the association between historical redlining and breast cancer screening prevalence, accounting for social vulnerability, neighborhood characteristics, and geographic access, and to characterize spatiotemporal screening patterns.

**Design, Setting, and Participants:**

This cohort study used Census tract–level data from 2016 to 2024 across the University of Kansas Cancer Center catchment area, including communities in Kansas and adjacent Missouri counties. The analytic sample included Census tracts with available Homeowners’ Loan Corporation (HOLC) grades A (indicating the least redlining) to D (indicating the most redlining). Statistical analysis was performed from July to December 2025.

**Exposures:**

HOLC grades; Social Vulnerability Index quintiles (with the first quintile indicating the lowest vulnerability and the fifth quintile indicating the highest); Census tract–level socioeconomic, housing, and transportation indicators; and distance to the nearest mammography facility.

**Main Outcomes and Measures:**

Census tract–level breast cancer screening prevalence from the Centers for Disease Control and Prevention’s PLACES database, reported as odds ratios (ORs) with 95% credible intervals (CrIs).

**Results:**

A total of 1152 historically redlined Census tracts were analyzed. Tracts were categorized by HOLC grades A (n = 27), B (n = 99), C (n = 423), and D (n = 603). The median (IQR) screening prevalence was highest in grade A tracts (79.6% [79.0%-80.0%]) and lowest in grade D tracts (75.2% [71.7%-79.2%]). Models demonstrated substantial spatial and temporal dependence with geographic clustering and localized variability. Compared with grade A tracts, grade C (OR, 0.94; 95% CrI, 0.90-0.99) and grade D tracts (OR, 0.94; 95% CrI, 0.89-0.99) had lower screening prevalence after adjustment. The highest Social Vulnerability Index quintile was associated with increased screening odds (OR, 1.08; 95% CrI, 1.01-1.14). Lower educational attainment (OR, 0.94; 95% CrI, 0.92-0.96) and higher mobile home prevalence (OR, 0.98; 95% CrI, 0.97-1.00) were associated with lower screening odds. Residual spatial heterogeneity persisted (711 of 1152 tracts [61.7%] excluding the null). Screening peaked in 2018 to 2019, stabilized through 2023, and declined in 2024, with most areas remaining below the Healthy People 2030 target of 80.3%.

**Conclusions and Relevance:**

This study found that historical redlining was associated with lower breast cancer screening prevalence independent of contemporary social vulnerability, neighborhood conditions, and geographic access. These findings support sustained, place-based strategies addressing structural and socioeconomic barriers to improve screening uptake and progress toward national screening targets.

## Key Points

**Question:**

Is historical redlining associated with breast cancer screening prevalence in a cancer center catchment area after accounting for social vulnerability, neighborhood socioeconomic conditions, and geographic access?

**Findings:**

In this cohort study of 1152 historically redlined Census tracts, tracts with Homeowners’ Loan Corporation grades C and D (representing more redlining) had significantly lower odds of receiving breast cancer screening than grade A tracts (representing the least redlining). Substantial spatial clustering and temporal dependence persisted after adjustment, and most neighborhoods remained below national screening targets.

**Meaning:**

The finding that historical redlining was associated with breast cancer screening disparities supports place-based strategies that address persistent socioeconomic barriers.

## Teaser

This cohort study examines the association of historical neighborhood redlining with breast cancer screening uptake in a cancer center catchment area in the Midwest.

## Introduction

Breast cancer is one of the most common cancers among women and remains a leading cause of cancer-related mortality. In the US, it is ranked as the second leading cause of cancer death among women, after lung cancer.^1,2,3^ Although the burden remains substantial, outcomes have improved considerably over time; breast cancer death rates declined by approximately 40% between 1989 and 2017, reflecting advances in treatment and earlier detection.^4^ Mammography screening is therefore considered a cornerstone of breast cancer control, supporting timely diagnosis and more effective intervention.^5,6^ However, screening prevalence is not evenly distributed across communities, and recent evidence has shown that social and structural barriers, such as transportation and other unmet social needs, continue to hinder screening utilization even when services are available.^7,8,9^

Historically produced inequities in neighborhood infrastructure and access to services may contribute to contemporary screening patterns. One key structural mechanism is the Homeowners’ Loan Corporation (HOLC) residential security grading system, commonly referred to as historical redlining, which classifies neighborhoods from “best” (grade A) to “hazardous” (grade D) based on perceived mortgage lending risk and investment potential.^10^ These classifications contributed to long-term disinvestment and reduced neighborhood resources in communities labeled as declining or hazardous. These historical patterns are linked to persistent differences in cancer-related outcomes and contemporary inequities in health, including breast cancer outcomes.^9,10,11,12,13,14,15^ Contemporary neighborhood vulnerability is also shaped by present-day socioeconomic and demographic conditions and is commonly captured by the Centers for Disease Control and Prevention (CDC) and Agency for Toxic Substances and Disease Registry Social Vulnerability Index (SVI), which is based on percentile rankings, with higher values indicating greater vulnerability.^16,17,18,19^ While the SVI captures current disadvantage across multiple domains, the enduring imprint of historical redlining may not be fully represented. In addition, geographic access to screening remains an important pathway, as distance to mammography facilities can influence preventive care use.^20,21,22^

National benchmarks highlight both progress and remaining gaps. Healthy People 2030 set a target of 80.3% for the proportion of women aged 50 to 74 years who are up to date with breast cancer screening, while the most recent (2023) national estimate was 79.8%.^23^ The 2024 Behavioral Risk Factor Surveillance System estimates from State Cancer Profiles showed that mammography use among women aged 50 to 74 years was 77.4% in Kansas and 79.5% in Missouri, compared with a US median of 78.5%.^2,4^ Thus, Kansas remains below both the 79.8% national estimate and the 80.3% Healthy People 2030 target, while Missouri is close to the 79.8% estimate but still below the 80.3% target.^24^ The Healthy People 2030 target is used as the primary national public health benchmark for the present analysis because it provides a federal population-level screening goal aligned with the eligible age group used in the breast cancer screening measure of the CDC PLACES project. These issues are particularly relevant for the University of Kansas Cancer Center (KUCC) catchment area, which spans communities across Kansas and adjoining counties in Missouri. For breast cancer prevention planning, it is important not only to evaluate whether historical redlining and social vulnerability are associated with screening but also to understand how screening prevalence is patterned across space and time. Census tract–level screening estimates commonly exhibit spatial dependence and year-to-year temporal correlation; analyses that ignore these features may underestimate uncertainty and miss meaningful geographic clustering.^25^ A preliminary analysis of the catchment area identified substantial geographic variation in breast cancer screening prevalence, with tract-level screening rates ranging from 56.9% to 84.5%, higher screening rates in urban centers, and lower screening rates in rural areas of southern and western Kansas. These geographic differences motivated our use of bayesian spatiotemporal modeling to account for spatial and temporal dependence in tract-level screening prevalence.

Despite increasing evidence linking historical redlining to cancer screening disparities, key gaps remain. Prior studies often had a cross-sectional design, and it remains unclear whether redlining is associated with breast cancer screening rates in the catchment area independent of present-day SVI, neighborhood conditions, and geographic access to mammography services. This study aimed to examine the association between historical redlining and breast cancer screening prevalence, accounting for social vulnerability, neighborhood characteristics, and geographic access, and to characterize spatiotemporal screening patterns. Specifically, we evaluated whether screening prevalence is lower in historically redlined Census tracts, particularly with HOLC grades C and D, and whether contemporary socioeconomic, housing, and transportation conditions help explain these disparities. The findings may generate place-based evidence to support cancer center outreach, community engagement, navigation, and resource allocation for neighborhoods where structural and socioeconomic barriers continue to limit progress toward national screening targets.

## Methods

### Study Design and Data Sources

A longitudinal ecological study was conducted to examine Census tract–level breast cancer screening rates in the KUCC catchment area (123 counties, including 105 in Kansas and 18 in Missouri) from 2016 to 2024. In accordance with University of Kansas Medical Center institutional policy, this study was exempt from ethics review and informed consent requirement because it used publicly available, deidentified, aggregate data. We followed the Strengthening the Reporting of Observational Studies in Epidemiology (STROBE) reporting guideline for cohort studies.

Annual Census tract–level breast cancer screening prevalence estimates were obtained from the CDC PLACES database and linked to historical redlining and present-day neighborhood characteristics via the Census tract geographic identifier using publicly available datasets.^20^ The CDC PLACES breast cancer screening measure represents the estimated percentage of women aged 50 to 74 years who report receiving a mammogram within the recommended screening interval and should not be interpreted as annual mammography use or individual-level adherence to yearly screening.

Historical redlining was measured using digitized HOLC residential security grades (grades A to D) from the Mapping Inequality project.^10^ HOLC grade assignment was harmonized to 2010 Census tract boundaries because the HOLC tract–based linkage file and historical redlining overlap calculations were based on 2010 tract geography. Digitized HOLC polygons were intersected with 2010 Census tract boundaries, and the percentage of each Census tract area overlapping each HOLC grades A to D was calculated. For tracts overlapping multiple HOLC grades, the final tract–level HOLC grade was assigned using the grade with the largest percentage area overlap. Present-day social context was characterized using the SVI, which was categorized into national quintiles, with the first quintile indicating the lowest vulnerability and the fifth quintile indicating the highest.^17^ Additional Census tract–level covariates captured socioeconomic conditions, household composition, racial and ethnic diversity and language status, and housing and transportation constraints (Table 1).^21^ The SVI was included as a composite measure of overall neighborhood vulnerability, whereas selected individual covariates were included to represent specific socioeconomic, housing, and transportation pathways that may link historical redlining to screening prevalence; conditional estimates for these covariates were interpreted cautiously because of conceptual overlap with the SVI. The screening outcome was measured annually from 2016 to 2024, whereas HOLC grade, SVI, neighborhood covariates, and mammography facility distance were treated as Census tract–level contextual measures that were fixed over the study period or measured using the closest available data source. Geographic access to screening was measured as straight-line distance (miles) from each centroid to the nearest Mammography Quality Standards Act–certified mammography facility using the US Food and Drug Administration facility database.^26^ Rurality was not included because the analytic sample comprised urban tracts only and lacked meaningful variation.

**Table 1.** Breast Cancer Screening Prevalence and Census Tract–Level Socioeconomic Characteristics by HOLC Redlining Grade

Characteristic	Census tracts, median (IQR)					*P* value^a^
	Overall (n = 1151)	HOLC redlining grade				
		A (n = 27)	B (n = 99)	C (n = 423)	D (n = 603)	
Breast cancer screening rate, %	76.3 (72.8-79.6)	79.6 (79.0-80.0)	78.2 (75.6-79.5)	77.1 (73.5-79.7)	75.2 (71.7-79.2)	<.001
SVI quintile, No. (%)						
1st, lowest	135 (11.7)	27 (100)	63 (63.6)	36 (8.5)	9 (1.5)	<.001
2nd	135 (11.7)	0	0	90 (21.3)	45 (7.5)	
3rd	138 (12.0)	0	0	63 (14.9)	75 (12.4)	
4th	295 (25.6)	0	18 (18.2)	127 (30.0)	150 (24.9)	
5th, highest	449 (39.0)	0	18 (18.2)	107 (25.3)	324 (53.7)	
Below 150% FPL	35 (19-46)	3 (2-6)	8 (3-30)	30 (19-39)	41 (30-51)	<.001
Unemployed	4.8 (2.7-7.7)	1.3 (0-1.3)	2.6 (1.6-7.4)	4.3 (2.7-6.3)	6.2 (3.3-8.7)	<.001
Housing cost burdened	31 (24-42)	8 (6-15)	19 (13-34)	29 (24-41)	35 (28-43)	<.001
No high school diploma	14 (7-23)	0 (0-2)	3 (1-4)	11 (3-18)	18 (10-26)	<.001
No health insurance	16 (10-22)	0 (0-3)	5 (4-12)	18 (10-20)	17 (12-26)	<.001
Aged ≥65 y	13 (8.8-16.3)	17 (16-28.2)	15 (12.1-18)	13.2 (10.4-16)	11.5 (7.3-16.1)	<.001
Aged ≤17 y	24 (18-29)	29 (13-31)	21 (19-27)	22 (12-27)	25 (21-31)	<.001
Civilians with a disability	14.4 (9.9-17.3)	5.4 (2.5-6.7)	9.1 (5.1-14.0)	14.9 (10.5-17.1)	14.9 (11.7-18.3)	<.001
Single-parent households	6.5 (2.7-11.7)	2.6 (0.8-3.8)	2.9 (0.8-5.8)	4.4 (1.8-7.7)	9.1 (5.2-14.1)	<.001
Limited English proficiency	2 (0-8)	0 (0-0)	1 (0-2)	2 (0-5)	3 (1-12)	<.001
Racially and ethnically minoritized	66 (30-84)	9 (7-14)	23 (12-62)	63 (28-85)	76 (47-87)	<.001
Living in multiunit structures	7 (1-22)	1 (0-12)	1 (0-9)	9 (2-22)	10 (2-26)	<.001
Living in mobile homes	0 (0-0.5)	0.0 (0.0-0.70)	0.0 (0.0-0.0)	0.0 (0.0-0.0)	0.0 (0.0-1.1)	<.001
Living in crowded conditions	2.2 (0.4-4.4)	0.0 (0.0-2.4)	0.5 (0.0-2.6)	1.4 (0.0-3.4)	2.5 (1.0-5.1)	<.001
Living in group quarters	0.3 (0-2)	0.0 (0.0-0.0)	0.2 (0.0-0.9)	0.5 (0.0-2.7)	0.2 (0.0-1.8)	<.001
No vehicle	9 (4-18)	1 (0-2)	2 (1-8)	7 (4-17)	14 (5-20)	<.001

Abbreviations: FPL, federal poverty level; HOLC, Homeowners’ Loan Corporation; SVI, Social Vulnerability Index.

^a^ Calculated with Kruskal-Wallis tests for continuous variables and with Pearson χ^2^ tests for categorical variables.

A total of 9071 Census tracts were initially considered for inclusion across the study period. Of these tracts, 2519 were identified based on the availability of HOLC grades (A to D). Among these, 224 tracts were excluded because of missing analytic variables, yielding 2275 tracts. To ensure 1 record per Census tract in each year, duplicate tract-year records were resolved after spatial linkage between Census tract boundaries and HOLC polygons. These duplicates occurred when a Census tract overlapped more than 1 HOLC polygon or grade. For each tract-year, the percentage of tract area overlapping each HOLC grade was calculated, and the tract was assigned the HOLC grade with the largest percentage area overlap. After this assignment, duplicate tract-year records were collapsed to 1 record per census tract-year. This process resulted in a final analytic sample of 1152 Census tracts for the bayesian spatiotemporal β regression analyses. The data cleaning and selection process is shown in eFigure 1 in Supplement 1. The initial count reflected tract-year records from 2016 to 2024 rather than unique Census tracts. A formal comparison of HOLC-graded and non–HOLC-graded tracts was not performed because HOLC maps were created primarily for historically urban areas and do not cover the full catchment area. Therefore, findings were interpreted as applicable to historically HOLC-graded urban tracts rather than all Census tracts in the catchment area.

### Statistical Analysis

Descriptive characteristics were summarized by HOLC grade using medians and IQRs or counts and percentages. Group differences were assessed using Kruskal-Wallis tests for continuous variables and Pearson χ^2^ tests for categorical variables.

Breast cancer screening prevalence, modeled as a proportion bounded between 0 and 1, was analyzed using bayesian spatiotemporal β regression with a logit link.^27,28^ The model included fixed effects for HOLC grade; SVI quintile; Census tract–level socioeconomic, housing, and transportation covariates; and mammography facility distance, along with intrinsic conditional autoregressive (ICAR) spatial random effects, first-order autoregressive (*AR*[1]) temporal random effects, and space-time interaction terms.^29,30,31,32,33^ Fixed-effect coefficients were exponentiated and reported as odds ratios (ORs) with 95% credible intervals (CrIs). For bayesian model results, statistical significance was defined as a 95% CrI excluding 1.00 for ORs. Full model equations, adjacency definitions, prior distributions, and computation details are provided in eMethods 1 in Supplement 1.^34,35^ Models were fit in R using the NIMBLE package (R Project for Statistical Computing), with 3 Markov Chain Monte Carlo of 10 000 iterations each, a 2000-iteration burn-in, and thinning of 2, yielding 12 000 posterior samples for inference.^36,37^ Posterior ICAR spatial random effects were exponentiated and mapped as tract-level spatial ORs. Tracts were classified as higher than expected, lower than expected, or not significant based on whether the 95% CrI for the tract-specific spatial OR was entirely above 1.0, entirely below 1.0, or crossed 1.0. To decompose the association between historical redlining and breast cancer screening prevalence into direct and indirect components, we conducted a tract-level multiple-mediator analysis restricted to 2024 within a structural equation modeling framework.^38,39,40,41^ The exposure contrasts HOLC grade D with grades A to C, and screening prevalence was modeled on the logit scale. Mediators included tract-level socioeconomic, housing, and transportation indicators (Table 1), and indirect associations were estimated as the product of the exposure-mediator and mediator-outcome paths, with uncertainty quantified using nonparametric bootstrap resampling.^40,42,43,44,45^ Corresponding 95% CIs were reported, and intervals excluding 0 were interpreted as evidence of an indirect association.

Two-sided *P* < .05 was considered statistically significant. Data analysis was performed from July to December 2025 using R, version 4.5.2 (R Project for Statistical Computing). Full model equations, mediator definitions, and implementation details are described in eMethods 2 in Supplement 1.

## Results

The analytic sample included 1152 historically redlined Census tracts categorized by HOLC grades A (n = 27), B (n = 99), C (n = 423), and D (n = 603), corresponding to 2.3%, 8.6%, 36.7%, and 52.3% of tracts, respectively. Descriptive statistics for breast cancer screening prevalence and Census tract–level socioeconomic characteristics by redlining grade are presented in Table 1.

Overall, tests indicated that screening prevalence and neighborhood characteristics varied across HOLC grades (all with *P* < .001) (Table 1). Descriptively, median (IQR) breast cancer screening prevalence was highest among grade A tracts (79.6% [79.0%-80.0%]) and lowest among grade D tracts (75.2% [71.7%-79.2%]). Social vulnerability showed the opposite pattern: all 27 Grade A tracts (100%) were in the lowest SVI quintile, whereas 324 Grade D tracts (53.4%) were in the highest quintile. Table 1 also shows descriptive gradients across HOLC grades in poverty, unemployment, lack of health insurance, crowded housing, and lack of transportation.

To assess the association between historical redlining and breast cancer screening prevalence after accounting for SVI and other covariates, we fitted a bayesian spatiotemporal β regression model with ICAR spatial random effects, *AR*(1) temporal random effects, and a space-time interaction. Model specifications were compared using the deviance information criterion (DIC), and the model with the lowest DIC was retained for inference. Using this selected model, we observed substantial residual geographic heterogeneity, captured by the spatial random effect *u_s_*: 711 of 1152 tracts (61.7%) had 95% CrIs for *u_s_* excluding 0, indicating meaningful spatial clustering in screening prevalence beyond the measured covariates. Temporal dependence was also supported through the *AR*(1) temporal random effect *α_t_*, with the *AR*(1) coefficient ϕ having a 95% CrI of 0.623 to 0.995, consistent with persistent year-to-year correlation, and the space-time interaction γ*_st_* was significant for 305 of 1152 tract-year combinations (26.5%), indicating localized departures from the overall temporal pattern. The results are summarized with posterior means, posterior SDs, ORs, and 95% CrIs in Table 2. Because the β regression used a logit link for the mean screening prevalence, ORs were reported as the natural exponentiated form of the fixed-effect coefficients.

**Table 2.** Adjusted Associations of HOLC Redlining Grade and Census Tract–Level Characteristics With Breast Cancer Screening Prevalence

Covariate	β, mean (posterior SD)	OR (95% CrI)^a^
HOLC redlining grade		
A	1 [Reference]	1 [Reference]
B	−0.044 (0.034)	0.96 (0.89-1.02)
C	−0.064 (0.025)	0.94 (0.90-0.99)
D	−0.065 (0.026)	0.94 (0.89-0.99)
SVI quintile		
1st, lowest	1 [Reference]	1 [Reference]
2nd	0.060 (0.028)	1.06 (1.00-1.12)
3rd	0.056 (0.027)	1.06 (1.00-1.11)
4th	0.049 (0.029)	1.05 (0.99-1.10)
5th, highest	0.073 (0.031)	1.08 (1.01-1.14)
Below 150% FPL	−0.012 (0.012)	0.99 (0.97-1.01)
No high school diploma	−0.062 (0.014)	0.94 (0.92-0.96)
No health insurance	−0.016 (0.013)	0.98 (0.96-1.01)
Living in multiunit structures	0.009 (0.010)	1.01 (0.99-1.03)
Living in mobile homes	−0.017 (0.007)	0.98 (0.97-1.00)
Living in crowded conditions	0.006 (0.009)	1.01 (0.99-1.02)
No vehicle	−0.002 (0.009)	1.00 (0.98-1.02)
Nearest mammography facility, miles	−0.032 (0.019)	0.97 (0.93-1.01)

Abbreviations: CrI, credible interval; FPL, federal poverty level; HOLC, Homeowners’ Loan Corporation; OR, odds ratio; SVI, Social Vulnerability Index.

^a^ ORs were calculated by exponentiating the posterior means of the β coefficients from the logit-linked β regression model and describe associations with mean screening prevalence on the log-odds scale. Because this analysis used a bayesian model, inference was based on 95% CrIs rather than frequentist *P* values.

In the unadjusted bayesian spatiotemporal β regression model including HOLC grade only, grade B (OR, 0.98; 95% CrI, 0.87-1.11), grade C (OR, 0.97; 95% CrI, 0.85-1.11), and grade D (OR, 0.96; 95% CrI, 0.82-1.09) tracts had lower but nonsignificant odds of breast cancer screening compared with grade A tracts. In the adjusted bayesian spatiotemporal model, historical redlining was associated with breast cancer screening. Compared with HOLC grade A, lower odds of screening were observed for grades C (OR, 0.94; 95% CrI, 0.90-0.99) and grade D (OR, 0.94; 95% CrI, 0.89-0.99) tracts after adjustment for SVI; socioeconomic, housing, and transportation covariates; mammography facility distance; and spatial and temporal structures.

Because the primary objective was to estimate the association between HOLC grade and breast cancer screening after adjustment for contemporary neighborhood conditions, conditional associations for SVI and other covariates are presented in Table 2 but were not interpreted as primary findings. The highest SVI quintile was associated with increased screening odds (OR, 1.08; 95% CrI, 1.01-1.14). Lack of high school diploma (OR, 0.94; 95% CrI, 0.92-0.96) and higher mobile home prevalence (OR, 0.98; 95% CrI, 0.97-1.00) were associated with lower screening odds. Although some higher-screening areas appear to be located nearer mammography centers, distance to the nearest facility was not independently associated with screening prevalence after adjustment for redlining, SVI, neighborhood socioeconomic characteristics, and spatial structure. The model also identified meaningful temporal variation in screening prevalence across the study period: temporal random effects indicated screening increased from 2016 to 2017, peaked in 2018 to 2019, remained stable through 2020 to 2023, and declined in 2024 (eFigure 2 in Supplement 1), suggesting time-varying screening levels beyond redlining, SVI, and other covariates.

**Figure 1.**
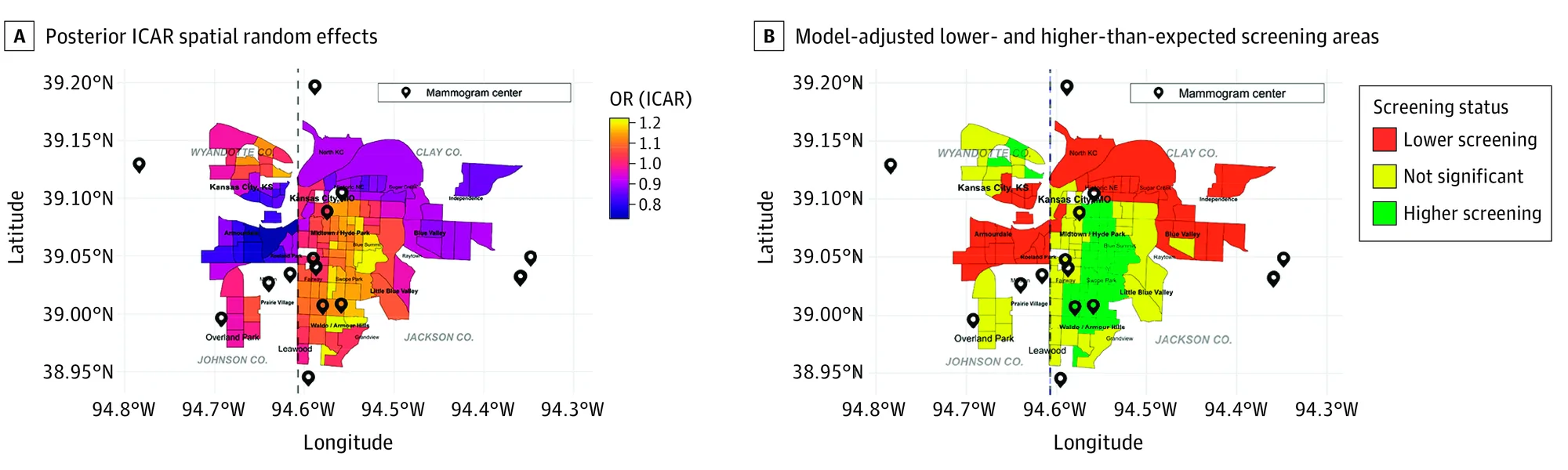
Maps of Model-Adjusted Spatial Patterns in Breast Cancer Screening Prevalence, With Mammography Centers Overlaid These values reflect model-adjusted residual spatial patterns, not crude screening prevalence. A, Posterior intrinsic conditional autoregressive (ICAR) spatial random effects are expressed as odds ratios (ORs). Values greater than 1.0 indicate higher-than-expected screening after model adjustment, whereas values less than 1.0 indicate lower-than-expected screening after model adjustment. Lighter or yellow areas represent higher residual screening, and darker or purple areas represent lower residual screening. B, Model-adjusted screening areas are based on posterior ICAR spatial random effects. Lower screening indicates Census tracts with significantly lower-than-expected screening after model adjustment, defined by a 95% credible interval (CrI) for the tract-specific spatial OR entirely below 1.0. Higher screening indicates Census tracts with significantly higher-than-expected screening after model adjustment, defined by a 95% CrI entirely above 1.0. Not significant indicates tracts with CrIs crossing 1.0. Screening status was consolidated over the 2016 to 2024 study period.

Beyond temporal patterns, the model revealed substantial residual geographic heterogeneity across the catchment area (Figure 1). The continuous posterior ICAR spatial ORs are shown in Figure 1A, and the corresponding classifications of lower-than-expected, higher-than-expected, and not significant screening areas are shown in Figure 1B. Lower-than-expected screening clusters were observed in areas such as Independence, Blue Valley, North Kansas City, and Armourdale. In contrast, higher-than-expected screening clusters appeared in Midtown/Hyde Park, Waldo/Armour Hills, and Prairie Village. Some lower-than-expected screening clusters were located near mammography facilities, suggesting that proximity alone may not explain these patterns.

**Figure 2.**
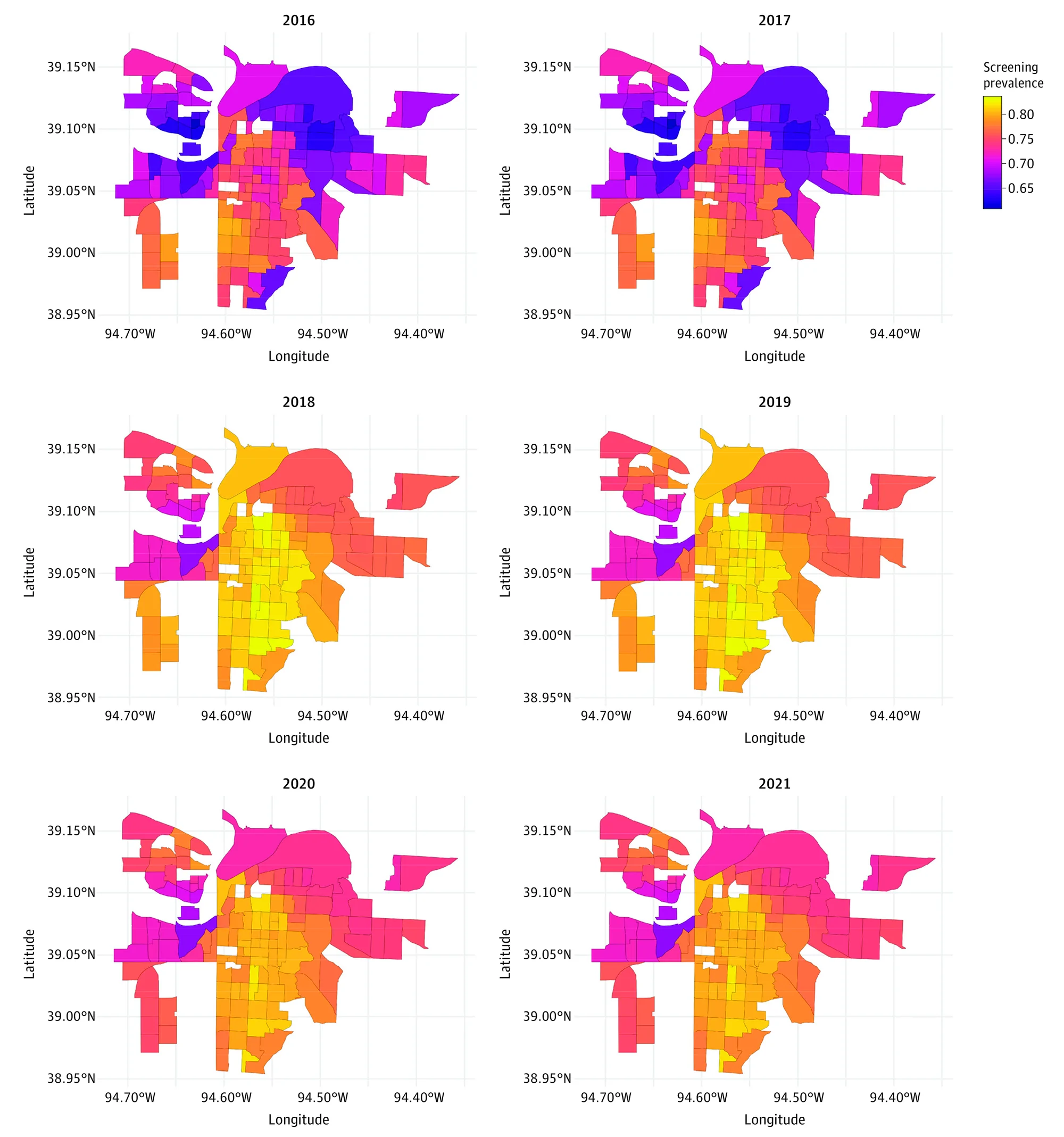
Maps of Year-Specific Model-Estimated Breast Cancer Screening Prevalence by Census Tract From 2016 to 2021

**Figure 3.**
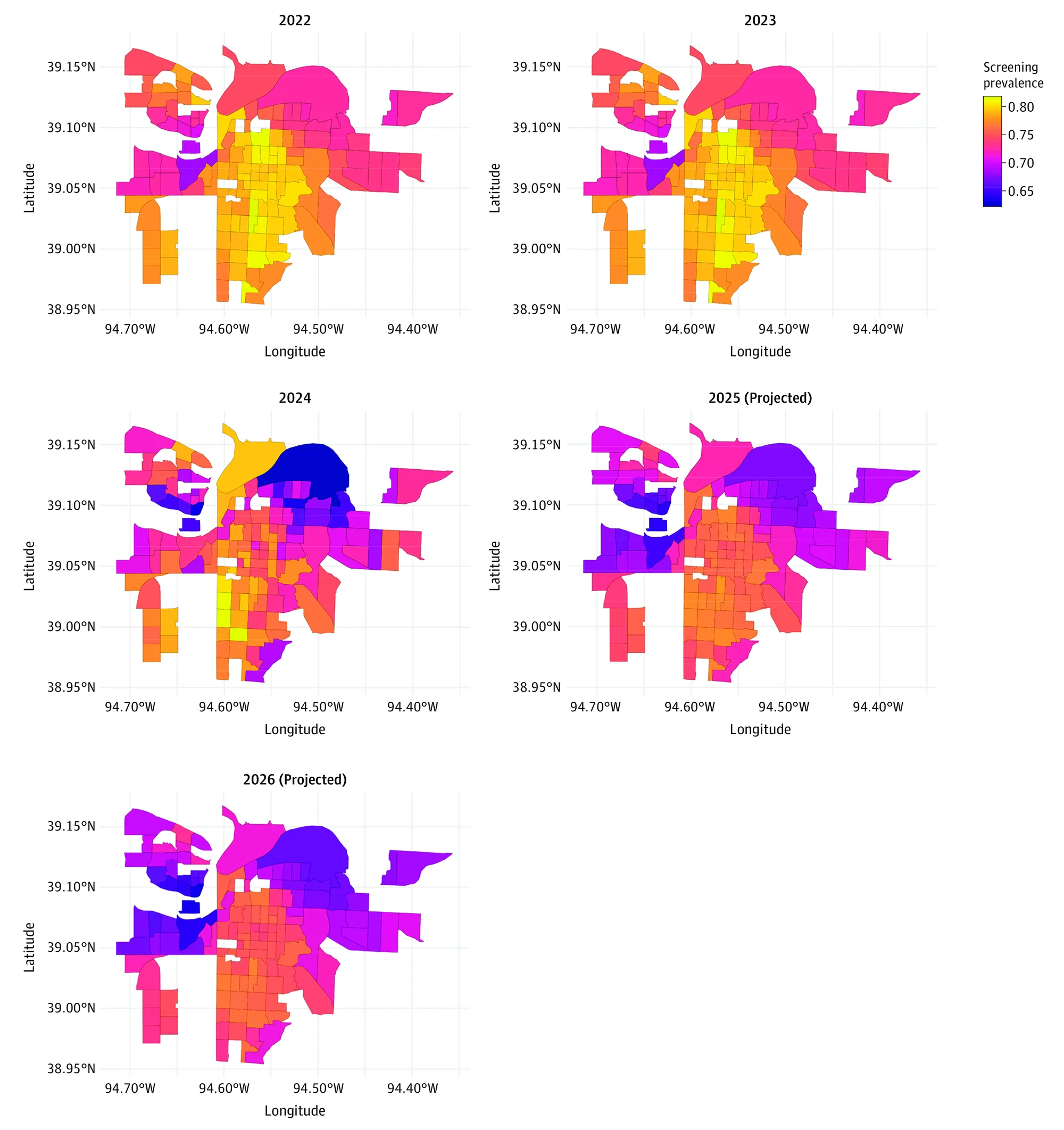
Maps of Year-Specific Model-Estimated Breast Cancer Screening Prevalence by Census Tract From 2022 to 2026 Projected values are shown for 2025 and 2026.

To characterize spatiotemporal patterns in breast cancer screening and inform planning, Figure 2 and Figure 3 present year-specific model-estimated screening prevalences for 2016 to 2024, along with short-term projections for 2025 and 2026. Screening prevalence ranged from 60.2% (693 of 1152) to 83.7% (964 of 1152). Model-estimated screening prevalence closely agreed with observed tract-level screening prevalence (Pearson *r* = 0.999), indicating close agreement between observed and model-estimated values. Estimated screening prevalence increased from 2017 to 2018, remained relatively stable through 2023, and declined in 2024. Despite these overall shifts, spatial disparities persisted: the same areas repeatedly showed lower estimated screening prevalence across years after adjustment for redlining, SVI, and other tract-level covariates. Model-based projections for 2025 and 2026 indicated that these lower-screening prevalence patterns may continue, including parts of Wyandotte County and eastern Jackson County, supporting geographically targeted outreach and resource allocation. Nationally, the Healthy People 2030 breast cancer screening target is 80.3%. Using 80.3% as a benchmark for progress toward the 2030 goal, each tract-year was classified as at or above the target (≥80.3%) vs below the target (<80.3%) (eFigure 3 in Supplement 1). Across 2016 through 2024, most tract-years in the study area (972 of 1152 [84.4%]) were below this benchmark, indicating that many neighborhoods remain behind the national 2030 screening target. These findings suggested that broad catchment-area efforts may be needed to improve screening overall, while spatial patterns may help identify neighborhoods where additional outreach, navigation, and structural supports should be prioritized.

To identify pathways linking historical redlining to screening prevalence in 2024, we conducted a tract-level multiple-mediator structural equation modeling comparing HOLC grade D vs grades A to C (eTable and eFigures 4 and 5 in Supplement 1). SVI was not included because it was a composite neighborhood vulnerability measure that overlapped with several individual socioeconomic, housing-, and transportation-related mediators considered separately in the analysis. The total association was explained primarily through socioeconomic mediators, with the largest contributions from poverty (44.5%; 95% CI, 22.7%-76.1%) and lower educational attainment (25.5%; 95% CI, 6.7%-54.0%), while lack of health insurance contributed minimally (1.4%; 95% CI, −11.0% to 15.1%). These findings suggest that socioeconomic conditions, particularly poverty and educational barriers, may be important intervention targets in historically redlined neighborhoods.

## Discussion

Breast cancer screening is essential for early detection, yet screening prevalence remains uneven across communities. Historically redlined neighborhood conditions may still be associated with access to care and use of preventive services. These findings suggest that historical redlining remains relevant to contemporary breast cancer screening patterns and may help identify neighborhoods where sustained screening outreach is needed.

In the bayesian spatiotemporal modeling, historical redlining was associated with lower contemporary breast cancer screening prevalence, with tracts graded C and D showing approximately 6% lower odds (grade C and D tracts’ OR, 0.94) than grade A tracts. Although the absolute difference in median screening rate between HOLC grade A and grade D tracts was modest (79.6% vs 75.2%), this 4.4 percentage–point difference may be meaningful at the population level when applied across neighborhoods and repeated screening cycles. Lower screening prevalence may represent missed opportunities for early detection, particularly in communities where socioeconomic barriers, transportation challenges, and unmet social needs may also delay diagnostic follow-up. Therefore, the observed difference was interpreted as a public health signal of persistent place-based screening disadvantage rather than as an individual-level estimate of late-stage diagnosis risk. These findings may also have implications for other screening-detected cancers, including cervical, colorectal, lung, and prostate cancers. Similar place-based barriers, including socioeconomic disadvantage, housing and transportation constraints, limited access to preventive services, and residual neighborhood-level spatial random effects, may influence screening uptake or early detection for these cancers. Therefore, the spatial patterns identified in this study may help inform broader cancer prevention and control efforts in the catchment area, particularly for screening programs that require repeated preventive care, timely diagnostic follow-up, and sustained patient navigation. Substantial residual spatial heterogeneity persisted after accounting for SVI and tract-level covariates, indicating geographic clustering of lower screening prevalence.

Screening prevalence also showed robust year-to-year dependence. Posterior estimates peaked in 2018 to 2019 and declined in 2024, while the same areas consistently exhibited lower screening prevalence across years, suggesting persistent localized disparities. Although higher neighborhood vulnerability was often expected to be associated with lower screening rates in population-level analyses, the highest SVI quintile in our fully adjusted spatiotemporal model showed slightly higher odds of screening. This finding should be interpreted as a conditional association rather than an overall vulnerability gradient, because SVI was a composite index that overlapped with historical redlining and several tract-level socioeconomic, housing, and transportation variables included separately in the model.

Spatial patterns suggested that lower screening prevalence remained concentrated in selected neighborhoods even after adjustment for measured tract-level characteristics. We identified several contiguous clusters of lower screening prevalence (eg, Independence, Blue Valley, North Kansas City, and Armourdale), as well as clusters of higher screening prevalence (eg, Midtown/Hyde Park, Waldo/Armour Hills, and Prairie Village). Mammography facilities were located within or near some clusters of low screening prevalence, suggesting that proximity alone may not explain these disparities and supporting targeted, place-based outreach to address persistent local barriers.

To contextualize the findings, we compared Census tract–level screening prevalence with national benchmarks. The Healthy People 2030 screening target is 80.3%, and the most recent national estimate is 79.8%.^23^ In the analytic sample, screening prevalence ranged from 60.2% to 83.7%, and 972 of 1152 Census tracts (84.4%) were below the Healthy People 2030 target. These local shortfalls suggest that patients living in neighborhoods with lower screening prevalence may experience missed or delayed opportunities for early detection, particularly in communities in which socioeconomic barriers and limited access to preventive services may also affect diagnostic follow-up. These findings indicated that national means may mask substantial local shortfalls and support targeted outreach in neighborhoods that remain below screening targets. This pattern aligned with prior evidence that historically redlined neighborhoods were less likely to meet screening targets, particularly in areas of higher SVI. Similar studies in other geographic settings have linked historical redlining and neighborhood social vulnerability with lower cancer screening rates and poorer cancer-related outcomes.^2,18,19,21,46,47,48,49,50,51,52^

To better understand the mechanisms underlying the association between historical redlining and breast cancer screening patterns, we performed a mediation analysis for the most recent year (2024) to assess whether socioeconomic conditions help explain this association. Because SVI was a composite measure that overlapped with several individual socioeconomic, housing-, and transportation-related pathway variables, it was not included in the mediation model. Results of the mediation analysis suggested that the association between redlining and screening operates largely through socioeconomic pathways. Poverty and lower educational attainment accounted for the largest mediated contributions, whereas distance to the nearest mammography facility and other housing and transportation indicators contributed little when modeled jointly. This pattern was consistent with a structural disinvestment framework in which long-term neighborhood conditions may be associated with preventive care use through economic constraints, competing demands, and reduced access to enabling resources rather than proximity alone.^21,41^ The direction of these findings was clinically plausible given the central role of routine mammography in promoting earlier detection, as reflected in current screening recommendations.^53,54^

Taken together, these findings suggested that interventions to improve screening uptake may be most effective when prioritized in neighborhoods with sustained screening shortfalls and may also support broader improvements in catchment-area screening. Model-based projections should be interpreted as scenario-based planning guidance rather than definitive forecasts. Mammography facilities were located within or near several clusters of low screening prevalence, indicating that reducing geographic distance alone may be insufficient. These findings support public health and health system decision-makers in prioritizing resources for historically underscreened neighborhoods through outreach and navigation supports that reduce socioeconomic and logistical barriers.^21^ From a policy and programmatic perspective, the spatial patterns identified in this study support a place-based approach to cancer prevention and have been shared with KUCC Community Outreach and Engagement leadership to inform outreach planning, community-engaged programming, navigation support, transportation assistance, mobile mammography strategies, and resource allocation for neighborhoods with persistent screening shortfalls. Because several clusters of lower screening prevalence were located near mammography facilities, interventions should address not only geographic proximity but also socioeconomic barriers, trust, awareness, appointment completion, and timely diagnostic follow-up.

### Limitations

Several limitations should be considered. First, this ecological study used Census tract–level screening estimates rather than individual-level behavior; thus, individual inference was not possible and residual confounding may remain. Second, CDC PLACES provides model-based small-area estimates; smoothing and measurement error may attenuate associations, and uncertainty may not be fully captured. In addition, because CDC PLACES provides annual small-area estimates of screening prevalence, yearly trends were interpreted as changes in estimated tract-level prevalence rather than individual-level annual mammography use or adherence to yearly screening schedules. Third, HOLC grades were available for only a subset of Census tracts, which may limit generalizability to nongraded areas; rurality also could not be evaluated because the analytic sample comprised urban tracts only. Fourth, the access measure used straight-line distance from each tract’s centroid to the nearest mammography facility as a simplifying proxy for access. This metric does not reflect road-network travel time (eg, routing-based estimates such as Google Maps), transportation mode, traffic, appointment availability, facility capacity, or insurance acceptance; therefore, access may be misclassified, and the null association with distance should be interpreted cautiously. Finally, tract-level covariates may not capture within-tract heterogeneity or time-varying neighborhood change.

## Conclusions

In this longitudinal ecological study of historically HOLC-graded Census tracts in the KUCC catchment area, historical redlining was associated with lower breast cancer screening prevalence independent of present-day social vulnerability. Persistent geographic clustering and temporal variation in screening were observed, with scenario-based projections suggesting these disparities may persist despite the passage of time. These pathway findings should be interpreted as descriptive rather than causal. Together, these findings highlight enduring place-based inequities and support targeted, place-based strategies to advance progress toward national screening targets.

## Notes

Supplement 1. **eMethods 1.** Bayesian Spatiotemporal Beta Regression Model (ICAR Spatial + AR(1) Temporal Effects) **eMethods 2.** Multiple-Mediator Structural Equation Model (SEM) for 2024 (HOLC Grade D vs Grades A–C) **eFigure 1.** Data Cleaning and Census Tract Selection Flowchart for the Bayesian Spatiotemporal Beta Regression Analyses **eFigure 2.** Posterior Temporal Random Effects, 2016-2024 **eFigure 3.** Tract-Year Classification Relative to Healthy People 2030 Benchmark (≥80.3%), 2016–2024 **eTable.** Multiple-Mediator SEM: Indirect Effects and Relative Contributions, 2024 **eFigure 4.** Mediator-Specific Indirect Effects (Bar Plot), 2024 **eFigure 5.** Mediation Pathway Summary (Sankey Diagram), 2024

Supplement 2.
Data Sharing Statement
