# Supplementary material for: Historical Redlining and Spatiotemporal Patterns in Breast Cancer Screening
Source: JAMA Netw Open. 2026 Sep 2;9(9):e2630685. doi: 10.1001/jamanetworkopen.2026.30685 (PMC13539404; doi:10.1001/jamanetworkopen.2026.30685)
Supplement: Supplementary file 1 [file jamanetwopen-e2630685-s001.pdf]

# Supplemental Online Content

Rahman MA, Ratnayake I, Pepper S, et al. Historical redlining and spatiotemporal patterns in breast cancer screening. *JAMA Netw Open*. 2026;9(9):e2630685. doi:10.1001/jamanetworkopen.2026.30685

**eMethods 1.** Bayesian Spatiotemporal Beta Regression Model (ICAR Spatial + AR(1) Temporal Effects)

**eMethods 2.** Multiple-Mediator Structural Equation Model (SEM) for 2024 (HOLC Grade D vs Grades A–C)

**eFigure 1.** Data Cleaning and Census Tract Selection Flowchart for the Bayesian Spatiotemporal Beta Regression Analyses

**eFigure 2.** Posterior Temporal Random Effects, 2016-2024

**eFigure 3.** Tract-Year Classification Relative to Healthy People 2030 Benchmark (≥80.3%), 2016–2024

**eTable.** Multiple-Mediator SEM: Indirect Effects and Relative Contributions, 2024

**eFigure 4.** Mediator-Specific Indirect Effects (Bar Plot), 2024

**eFigure 5.** Mediation Pathway Summary (Sankey Diagram), 2024

This supplemental material has been provided by the authors to give readers additional information about their work.

© 2026 Rahman MA et al. *JAMA Network Open*.

**eMethods 1: Bayesian spatiotemporal beta regression model**

Let $y_{st} \epsilon$ (0,1) denote the annual census-tract breast cancer screening prevalence for tract $s = 1, \dots, S$ in year $t = 1, \dots, T$ (2016–2024). Since the outcome is proportion, we modeled screening prevalence using a beta distribution parameterized by mean $\mu_{st}$ and precision ϕ:

$$y_{st} \sim Beta(\mu_{st}\phi, (1 - \mu_{st})\phi),$$

where $\phi > 0$ controls dispersion around the mean.[26,27] The mean was linked to a linear component $\eta_{st}$ using the logit link:

$$logit\ (\mu_{st}) = \eta_{st.}$$

The linear component is decomposed into fixed-effects and multiple random-effect components:

$$\eta_{st} = \beta_0 + \boldsymbol{X}_{st}^T\boldsymbol{\beta} + u_s + \alpha_t + \gamma_{st}.$$

Here, $\beta_0$ is an intercept; $\boldsymbol{X}_{st}$ is the vector of tract-level covariates (including HOLC grade, SVI quintile, and additional neighborhood characteristics); and $\boldsymbol{\beta}$ are the corresponding regression coefficients. The term $u_s$ captures residual geographic clustering shared across years after accounting for covariates, $\alpha_t$ captures correlation across years shared across tracts (overall temporal dependence), and $\gamma_{st}$ captures tract-specific departures from the spatial and temporal patterns (localized deviations by year).

**Spatial random effects (ICAR prior)**

To account for residual spatial correlation among neighboring census tracts, we assigned the spatial effects $u_s$ an ICAR prior.[28,29] Let $\boldsymbol{u} = (u_1, \dots, u_s)^T$ denote the vector of tract-level spatial random effects. The ICAR prior smooths the spatial effects across neighboring tracts and is defined as

$$\boldsymbol{u} \sim ICAR(\tau_{spatial}, \boldsymbol{Q}),$$

where $\tau_{spatial}$ is a spatial precision parameter and $\boldsymbol{Q} = \boldsymbol{D} - \boldsymbol{W}$ is the intrinsic CAR precision matrix constructed from the adjacent matrix $\boldsymbol{W}$. The $S \times S$ adjacency matrix $\boldsymbol{W} = (w_{ij})$ is defined as

$$w_{ij} = \begin{cases} 1 & if\ tracts\ \ i\ and\ j\ are\ neighbors, \\ 0 & otherwise. \end{cases}$$

Here, $i$ and $j$ index census tracts $(i, j = 1, \dots, S)$. For each tract $i$, the number of neighbors is defined as

$$d_i = \sum_{j=1}^{S} w_{ij,}$$

And these values are collected into a diagonal matrix

$$\boldsymbol{D} = diag(d_1, d_2, \dots, d_S).$$

With this notation, the ICAR prior can also be written in conditional form. For each tract $i$,

$$u_i | \{u_j : j \neq i\}, \tau_{spatial} \sim N\left(\frac{1}{d_i} \sum_{j:w_{ij}=1} u_j, \left(\tau_{spatial} d_i\right)^{-1}\right),$$

that is, the spatial effect $u_i$ is normally distributed around the average of its neighboring tracts, $\frac{1}{d_i}\sum_{j:w_{ij}=1} u_j$, with variance $\left(\tau_{spatial} d_i\right)^{-1}$.[28] Larger values of $\tau_{spatial}$ induce stronger spatial smoothing, pulling each tract's effect more tightly toward the average of its neighbors, while smaller values allow greater local heterogeneity.

© 2026 Rahman MA et al. *JAMA Network Open*.

In joint form, the ICAR prior has density (up to a normalizing constant)

$$p(\boldsymbol{u}|\tau_{spatial}) \propto exp\left(-\frac{\tau_{spatial}}{2}\boldsymbol{u}^{\boldsymbol{T}}\boldsymbol{Q}\boldsymbol{u}\right),$$

Where the quadratic form can be expanded as

$$\boldsymbol{u}^{\boldsymbol{T}}\boldsymbol{Q}\boldsymbol{u} = \sum_{i=1}^{S}\sum_{j=1}^{S} w_{ij}\left(u_i - u_j\right)^2.$$

This representation highlights that the ICAR prior penalizes large differences between spatial effects of neighboring tracts, thereby encouraging spatial smoothness while still allowing for localized deviations when supported by the data.[29,32] Because $\boldsymbol{Q} = \boldsymbol{D} - \boldsymbol{W}$ is singular with rank $S - 1$, the ICAR prior is intrinsic and defined only up to an additive constant. To ensure identifiability in the regression model, we imposed a sum-to-zero constraint on the spatial random effects,

$$\sum_{i=1}^{S} u_i = 0,$$

so that the overall mean level of the outcome is captured by the intercept and fixed effects, and the spatial random effects represent relative deviations around that mean.

**Temporal random effects ($\boldsymbol{AR}$ $(\mathbf{1})$ prior)**

To capture smooth temporal evolution in screening over the study period, we modeled the year effects $\alpha_t$ using a $AR(1)$ prior:

$$\alpha_1 \sim N\left(0, \sigma_{\alpha_0}^2\right), \quad \alpha_t|\alpha_{t-1} \sim N(\rho\alpha_{t-1}, \sigma_\alpha^2), t = 2, \ldots, T,$$

where $\rho$ represents the temporal autocorrelation $(0 \leq \rho \leq 1)$ and $\sigma_\alpha^2$ controls year-to-year variability trends.[27,30] This structure induces correlation between adjacent years and yields a smooth temporal trend.

**Space-time interaction**

After accounting for marginal spatial $(u_s)$ and temporal $(\alpha_t)$ structure, some tracts may exhibit localized accelerations or declines over time. To allow for these tract-specific deviations, we included an additional space–time interaction term $(\gamma_{st})$.[30-32] In the primary specification, we modeled

$$\gamma_{st} \sim N\left(0, \sigma_\gamma^2\right),$$

where $\sigma_\gamma^2$ controls the magnitude of tract–year–specific deviations from the main spatial and temporal patterns.

**Prior specification and computation**

Mean-zero weakly informative priors are used to stabilize estimation and support convergence while allowing the data to dominate inference, particularly given standardized covariates and the hierarchical spatiotemporal structure. Fixed-effect coefficients are assigned $\beta_0 \sim N(0, 10^2)$ and $\beta_k \sim N(0, 5^2)$ for $k = 1, \ldots, K$.[34] The spatial ICAR component is implemented using a CAR-normal prior with a sum-to-zero constraint, with $\tau_{spatial} \sim Gamma(0.5, 0.0005)$.[29,35] Temporal random effects follow an $AR(1)$ process with $\rho \sim Uniform(0, 1)$, $\sigma_{\alpha 0} \sim Uniform(0, 5)$, and $\sigma_\alpha \sim Uniform(0, 5)$, where $\alpha_1 \sim N(0, \sigma_{\alpha 0})$ and $\alpha_t \sim N(\rho\alpha_{t-1}, \sigma_\alpha^2)$ for $t \geq 2$.[31] The space-time interaction is modeled as $\gamma_{st} \sim N(0, \sigma_{inter}^2)$ with $\sigma_{inter}^2 \sim Uniform(0, 5)$ and the beta precision parameter is assigned $\phi \sim Gamma(0.5, 0.0005)$.[27,32] Models are fit in R using *NIMBLE* with 3 Markov chain Monte Carlo (MCMC) chains of 10,000 iterations each, a 2,000-iteration burn-in, and thinning of 2, yielding 12,000 posterior samples for inference.[36,37]

© 2026 Rahman MA et al. *JAMA Network Open*.

**eMethods 2: Mediation analysis (SEM) model**

To focus on the most recent screening landscape, we conducted a tract-level multiple-mediator analysis restricted to 2024 within a regression-based SEM framework to decompose the association between redlining and screening prevalence into direct and indirect components.[37-40] Let $i = 1, \ldots, n$ index census tracts. For mediation, the exposure $X_i$ was defined by dichotomizing HOLC grade (Grade D vs A-C) to provide a single, interpretable disparity contrast for decomposition into direct and indirect effects.

The outcome $Y_i \in (0,1)$ (tract-level breast cancer screening prevalence) was modeled on the logit scale $Y_i^* = log\{Y_i/(1 - Y_i)\}$. Let $M_{ik}$ denote the $k^{th}$ mediator ($k = 1, \ldots, K$), and let $\boldsymbol{Z}_i$ denote the vector of covariates. For each mediator, we fit:

$$M_{ik} = \alpha_k + a_k X_i + \boldsymbol{\theta}_k^T \boldsymbol{Z}_i + \varepsilon_{\text{ik}}, \quad \varepsilon_{\text{ik}} \sim N(0, \sigma_k^2),$$

and the outcome model:

$$Y^* = \beta_0 + c_d X_i + \sum_{k=1}^{K} b_k M_{ik} + \boldsymbol{\delta}^T \boldsymbol{Z}_i + \varepsilon_{Y_i}, \ \ \varepsilon_{Y_i} \sim N(0, \sigma_Y^2),$$

where $a_k$ and $b_k$ are mediator-path coefficients.[41] Here, $c_d$ denotes the direct effect of redlining $(X)$ on screening $(Y^*)$ conditional on the mediators and covariates, and $a_k b_k$ denotes the mediator-specific indirect effects through mediator $k$. The total indirect effect was defined as $\sum_{k=1}^{K} a_k b_k$, and the total effects as $c_d + \sum_{k=1}^{K} a_k b_k$.[41,42] Inference for indirect effects used nonparametric bootstrap resampling with percentile 95% confidence intervals.[39,43] Analyses were implemented in R using lavaan.[44]

© 2026 Rahman MA et al. *JAMA Network Open*.

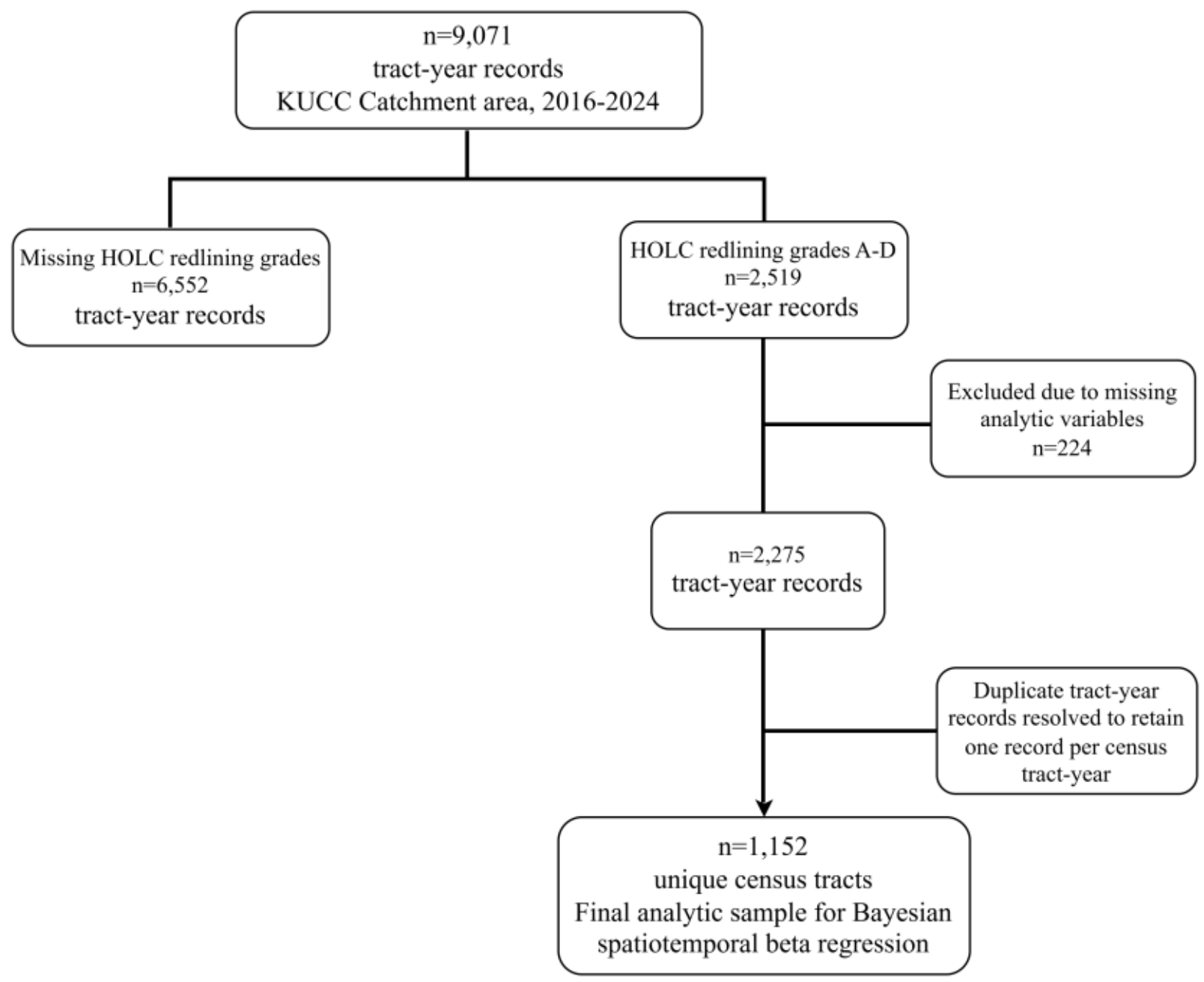


**eFigure 1**. Data cleaning and analytical census tract selection flowchart

The flow diagram shows the selection of tract-year records from the initial KUCC catchment-area dataset to the final Bayesian spatiotemporal analytic sample. Duplicate tract-year records occurred when a census tract overlapped more than one HOLC polygon or grade and were resolved by assigning the HOLC grade with the largest percentage area overlap.

© 2026 Rahman MA et al. *JAMA Network Open*.

Additional Bayesian model results, including posterior temporal effects, spatial effects, and model-based screening patterns, are provided to support the primary findings of the analysis.

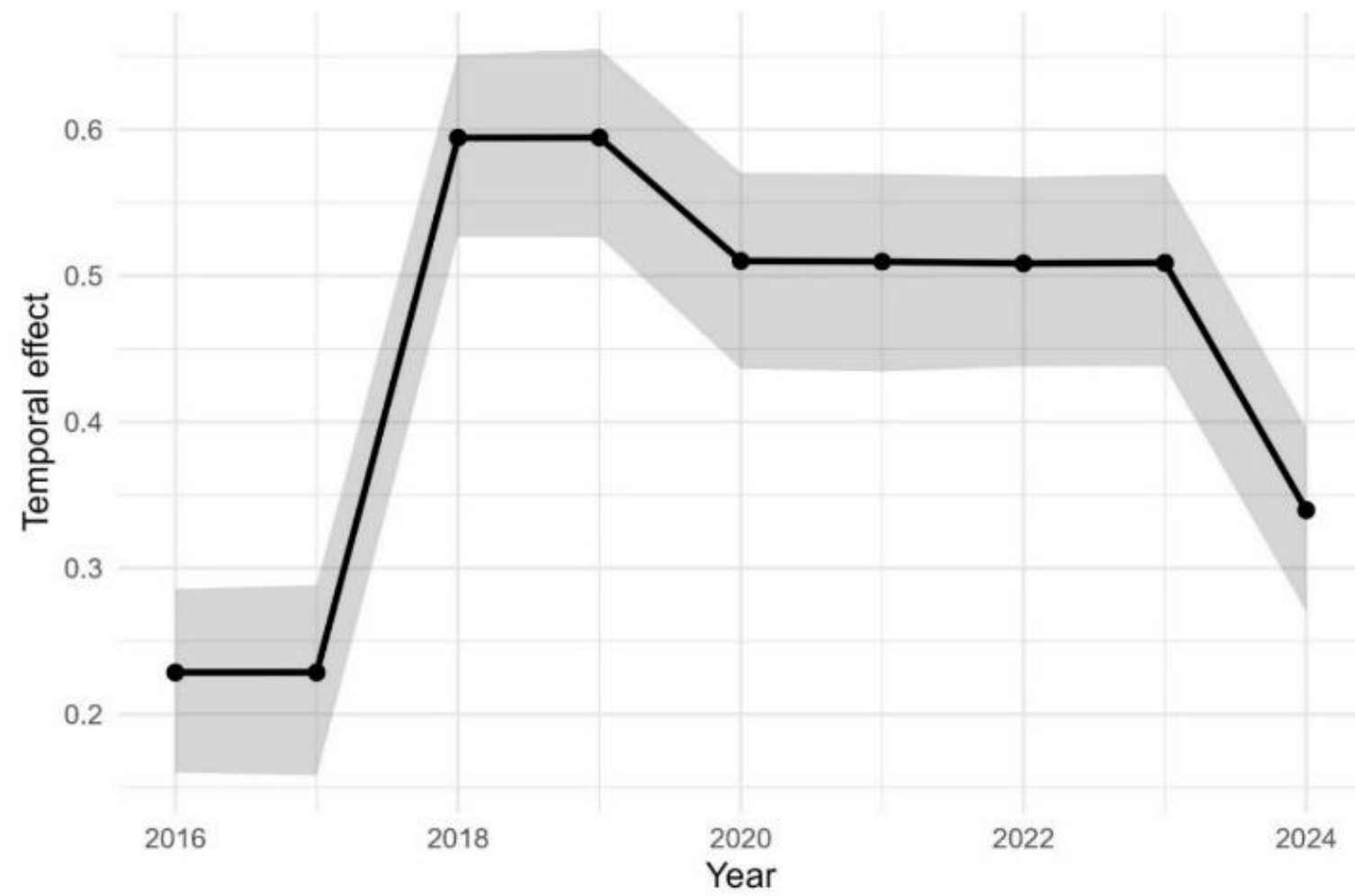


**eFigure 2. Posterior temporal random effects ($\alpha_t$) from the selected Bayesian spatiotemporal model (posterior mean with 95% credible intervals), 2016–2024.**

© 2026 Rahman MA et al. *JAMA Network Open*.

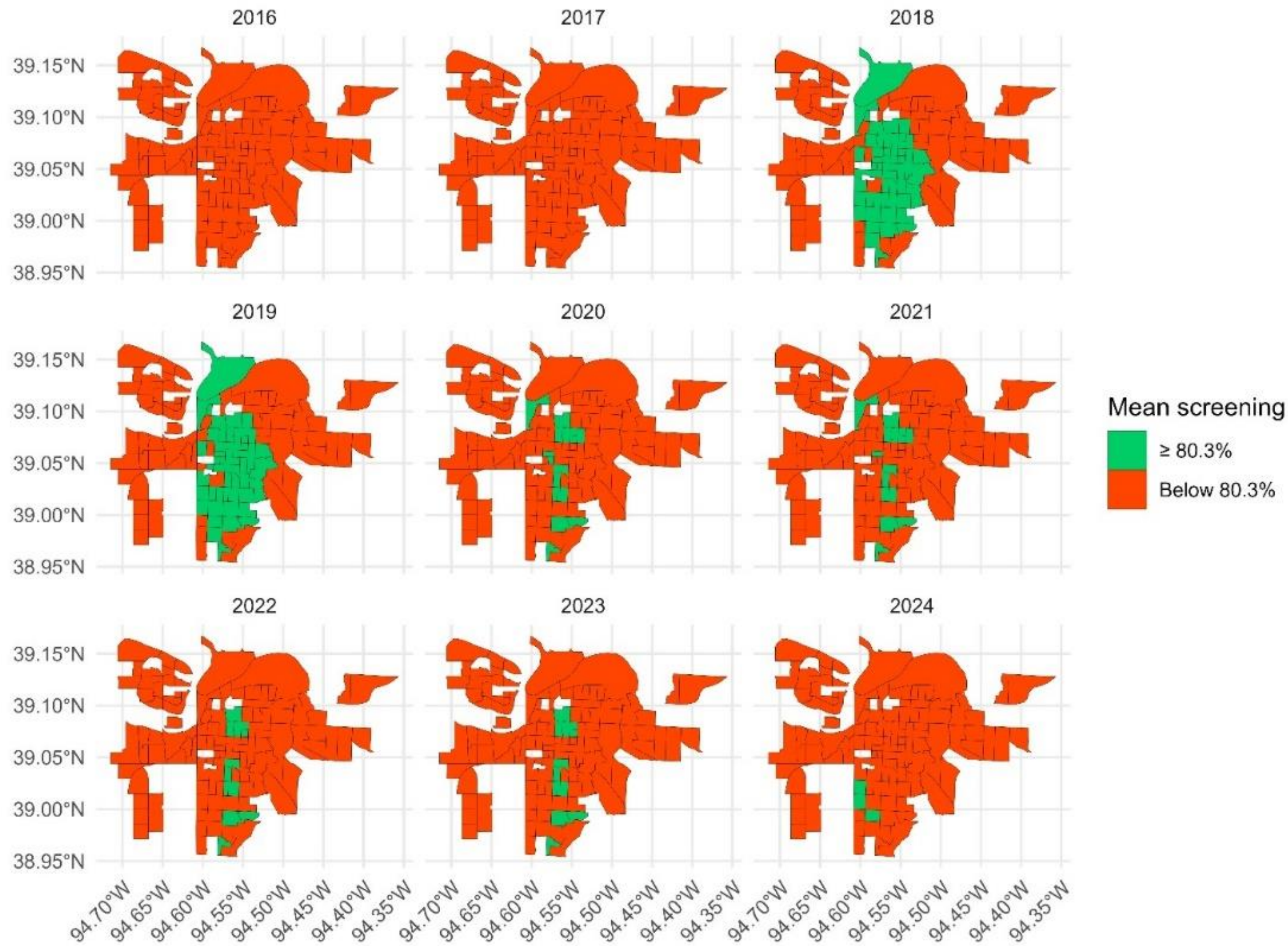


**eFigure 3. Year-specific spatial distribution of census tracts meeting the Healthy People 2030 breast cancer screening benchmark (≥80.3%), 2016–2024.**

© 2026 Rahman MA et al. *JAMA Network Open*.

Detailed results from the tract-level multiple-mediator SEM (HOLC Grade D vs Grades A–C) are provided below. Mediator-specific indirect effects and the proportion of the total association mediated were estimated using bootstrap percentile 95% confidence intervals.

**eTable. Mediation analysis of the association between historical redlining (HOLC Grade D vs Grades A–C) and census-tract–level breast cancer screening prevalence, 2024.**

| Mediator | Effect estimate (95% CI) | Relative effect (95% CI) |
|---|---|---|
| HOLC Grade D vs Grade A/B/C | | |
| Direct | -0.05 (-0.10, 0.01) | 35.2 (-9.6, 66.8) |
| Indirect | | |
| Poverty* | -0.06 (-0.10, -0.03) | 44.5 (22.7, 76.1) |
| No high school diploma* | -0.04 (-0.07, -0.01) | 25.5 (6.7, 54.0) |
| No health insurance | -0.00 (-0.02, 0.01) | 1.4 (-11.0, 15.1) |
| Multi-unit structures | 0.00 (-0.02, 0.02) | -2.0 (-17.7, 12.9) |
| Mobile homes | -0.00 (-0.01, 0.01) | 0.3 (-5.0, 10.6) |
| Crowded conditions | -0.00 (-0.01, 0.01) | 0.1 (-5.5, 8.2) |
| No vehicle | 0.01 (-0.00, 0.03) | -6.5 (-21.0, 1.9) |
| Nearest mammogram distance | -0.00 (-0.02, 0.01) | 1.3 (-6.8, 11.8) |
| Total indirect | -0.09 (-0.16, -0.04) | 64.8 (33.2, 109.6) |
| Total effect | -0.14 (-0.21, -0.08) | |
| *Bootstrap 95% confidence interval (CI) excludes 0. | | |

© 2026 Rahman MA et al. *JAMA Network Open*.

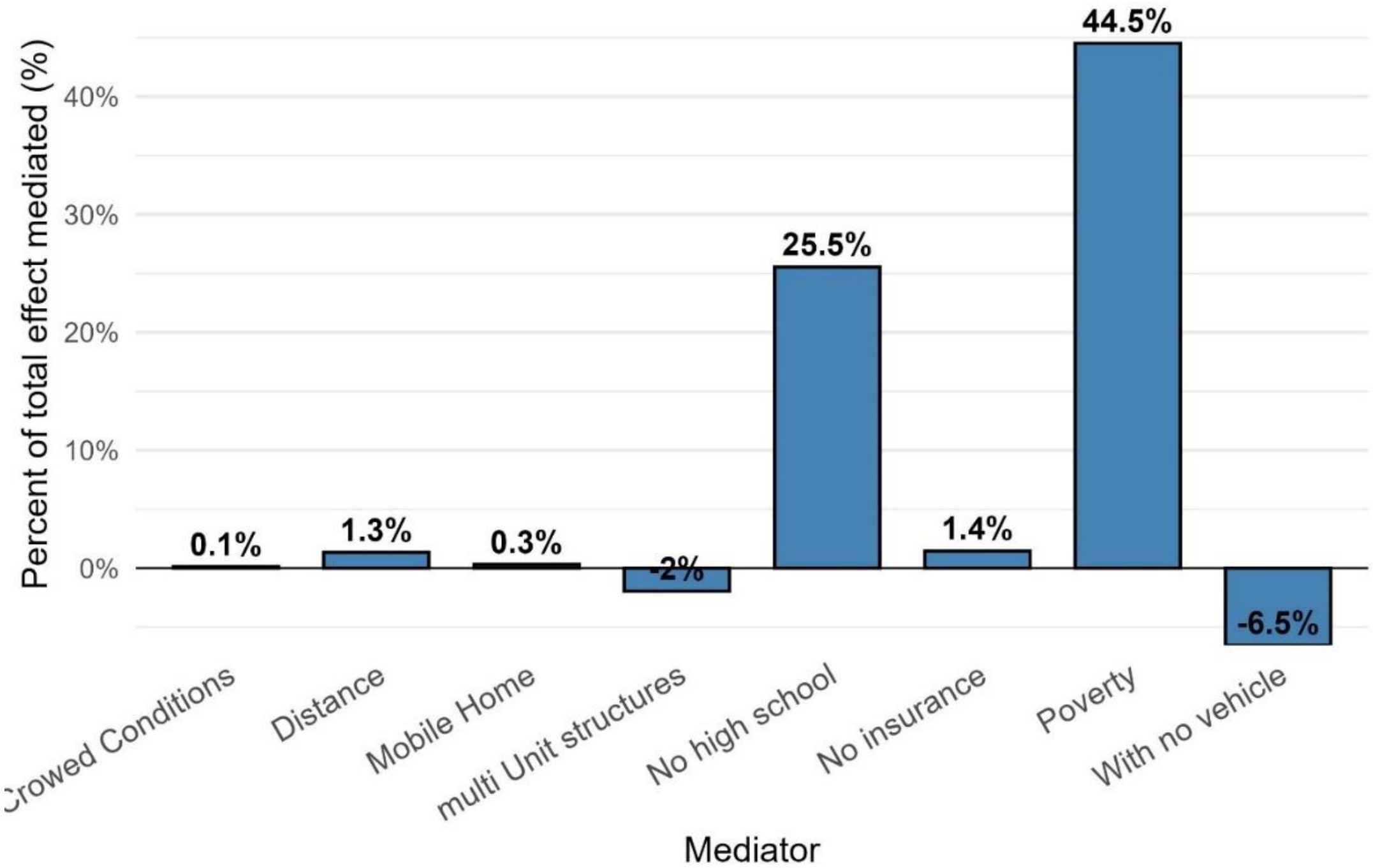


**eFigure 4. Bar plot of mediator-specific indirect effects and relative contributions to the total association between redlining and breast cancer screening prevalence, 2024.**

© 2026 Rahman MA et al. *JAMA Network Open*.

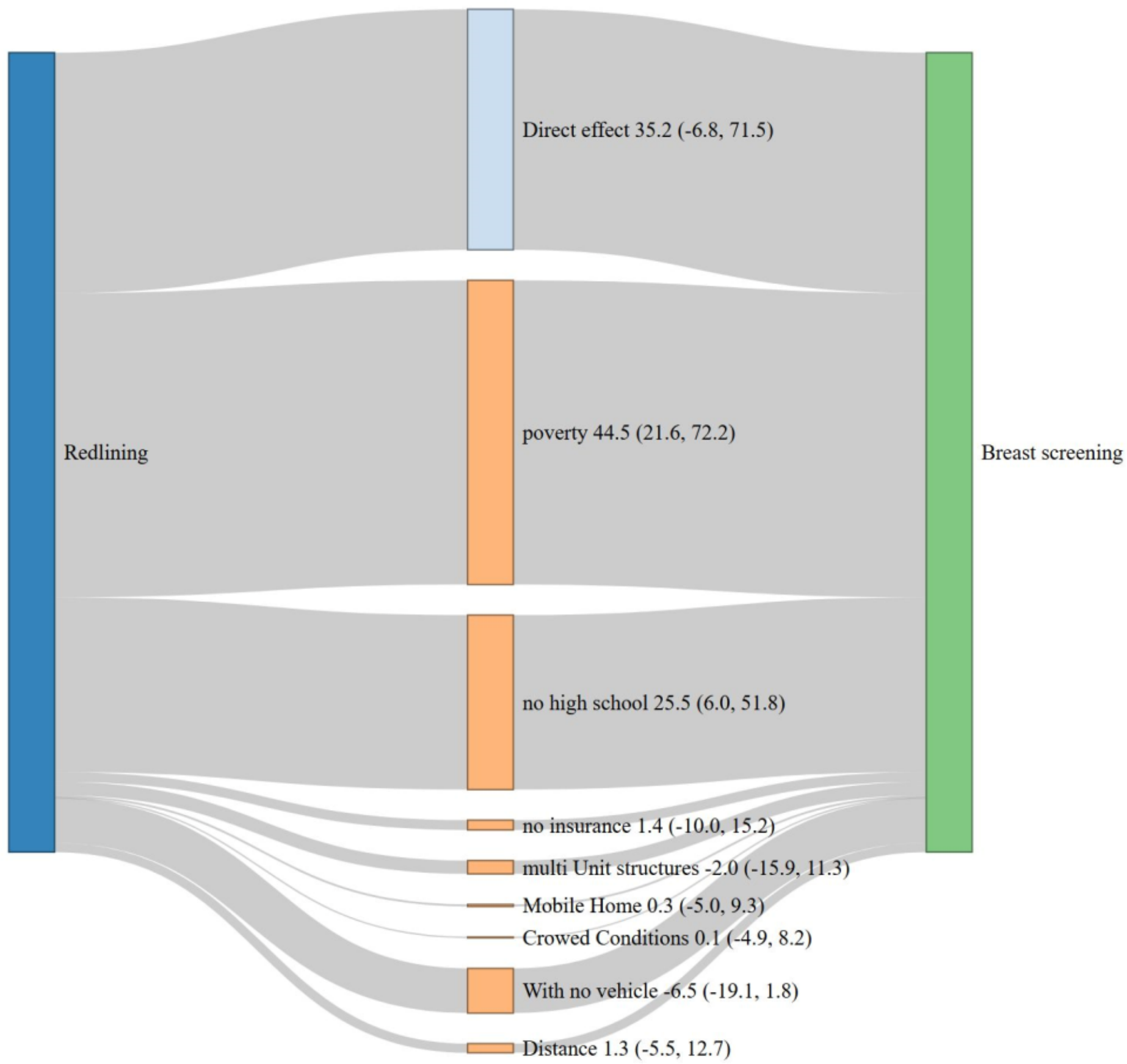


**eFigure 5. Sankey diagram summarizing direct and indirect pathways linking redlining to breast cancer screening prevalence through tract-level mediators, 2024.**

© 2026 Rahman MA et al. *JAMA Network Open*.
