# Supplementary material for: Historical Redlining and Spatiotemporal Patterns in Breast Cancer Screening
Source: JAMA Netw Open. 2026 Sep 2;9(9):e2630685. doi: 10.1001/jamanetworkopen.2026.30685 (PMC13539404; doi:10.1001/jamanetworkopen.2026.30685)
Supplement: Supplementary file 2 [file jamanetwopen-e2630685-s002.pdf]

Data Sharing Statement

Rahman. Historical Redlining and Spatiotemporal Patterns in Breast Cancer Screening. *JAMA Netw Open*. Published September 02, 2026. doi:10.1001/jamanetworkopen.2026.30685

Data
Data available: Yes
Data types: Data (not involving human participants)
How to access data: Publicly available data used in this study include CDC PLACES breast cancer screening estimates (https://www.cdc.gov/places), Mapping Inequality historical redlining maps/HOLC grades (https://dsl.richmond.edu/panorama/redlining), CDC/ATSDR Social Vulnerability Index (SVI) data (https://www.atsdr.cdc.gov/place-health/php/svi/svi-data-documentation-download.html), and the FDA Mammography Quality Standards Act (MQSA) certified facility database (https://www.fda.gov/radiation-emitting-products/mammography-information-patients/search-certified-facility).
When available: With publication

Supporting Documents
Document types: Statistical/analytic code, Other (please specify)
Additional Information: Will have the data set available via a git repository
How to access documents: Will also house the statistical code on the git repository
When available: With publication

Additional Information
Who can access the data: anyone requesting the data
Types of analyses: for any purpose
Mechanisms of data availability: with a signed data access agreement
